# Double-balloon enteroscopy in small bowel diseases: Eight years single-center experience in China

**DOI:** 10.1097/MD.0000000000005104

**Published:** 2016-10-21

**Authors:** Wen-Guo Chen, Guo-Dong Shan, Hong Zhang, Ming Yang, Lin L, Min Yue, Guang-Wu Chen, Qing Gu, Hua-Tuo Zhu, Guo-Qiang Xu, Li-Hua Chen

**Affiliations:** a Department of Gastroenterology; b Department of Endoscopy Center, The First Affiliated Hospital, College of Medicine, Zhejiang University, Hangzhou, Zhejiang Province, China.

**Keywords:** capsule endoscopy, diagnosis, double-balloon enteroscopy, endoscopic findings, small bowel disease

## Abstract

The aim of the study was to evaluate the diagnostic and therapeutic value of double-balloon entoroscopy (DBE) in small bowel diseases (SBDs) in China.

A retrospective review of 674 consecutive patients who underwent DBE between January 2007 and November 2015 was conducted. Patients were divided into 3 groups by age, young group (<45 years), middle-aged group (45–65 years), and elderly group (>65 years). Data were collected with regard to demographics, clinical, endoscopic findings, complications, diagnostic yield, and management.

A total of 729 DBE procedures were performed successfully in our series. More than 20 types of SBDs were found with the detection rate of 70.9%(517/729). The majority of patients were Crohn's disease (33.4%,225/674), followed by tumor (18.8%,127/674) and angioectasia (7.9%, 53/674). Endoscopic treatment was performed in 60 patients in which hemostasis (17,28.3%) and polypectomy (15,25%) were the predominant form of intervention used. Adverse events occurred in 6 patients (0.96%,6/729) including perforation, hemorrhage, aspiration pneumonia. No acute pancreatitis or other major complications occurred. Adenocarcinoma, GIST, and lymphoma were the most common tumor detected, the majority of tumors located in the jejunum (56.7%), The detection rate of angioectasia was also higher in the jejunum (54.7%),77.8% of Crohn's disease was located in the ileum. The positive rate of DBE in small bowel tumor and Crohn's disease were significantly higher than that of angioectasia (*P*<0.05). In young cohort, Crohn's disease (48.1%) was the most commonly diseases followed by tumor (10.4%) and nonspecific enteritis (7.1%). Yet in the elderly group, the majority of patients were tumor (27.6%); angioectasia (21.3%) was also detected frequently. The positive rate of capsule endoscopy was 75.44 %(202/268) which was a little high than DBE (67.9%, 182/268) (*P* > 0.05). The obscure gastrointestinal bleeding (OGIB) was the most common indication, and the diagnostic yield was 71.8%.

DBE is a useful diagnostic and therapeutic tool with high clinical practice value for the investigation of SBDs. With growing experience of endoscopist, we believe that DBE must be kept in mind as the first-line modality for suspected SBDs.

## 1 Introduction

The small bowel in the mid-GI tract has historically become blind spot to endoscopist due to the anatomy, location, and tortuosity. Small bowel diseases (SBDs) are less common in the entire digestive tract which lack specific signs and symptoms, its diagnosis, and management are formidable tasks for clinician.

Currently, the advent of capsule endoscopy (CE) and double balloon endoscopy (DBE) between 2000 and 2001 made the visualization of the entire small-bowel mucosa practical.^[1]^ They have been used in clinical practice worldwide. CE was considered a noninvasive imaging utilizing for screening the GI tract with minimum discomfort and developed into a first-line modality for the evaluation of SBDs. However, inability to obtain tissue samples and perform intervention limited its use. The shortcomings have been overcome through the application of DBE, first developed and reported by Yamamoto et al.^[2]^ Through the controlled enteroscope and overtube, we can achieve total small bowel evaluation combination antegrade and retrograde procedures. Therapeutic management, the qualitative diagnosis, and localization diagnosis of the lesion, all of these showed the unique advantage of DBE in the strategy for diagnosis and treatment of SBDs.

Up to now, more and more articles across the world addressed the value of DBE in SBDs. However, most of these studies were of small sample size or multicenter research, the majority of reported experience has come out of Japan, Europe.^[3,4]^ Meanwhile, disease detection rate in patients with different age groups was rarely mentioned. Between January 2007 and November 2015, >700 DBE examinations have been performed in our hospital. The aim of our study was mainly to assess its clinic value, comparison with CE, detection rate of different age groups, and so on.

## 2 Materials and Methods

### 2.1 Patients

All the 674 consecutive patients submitted to 729 DBE procedures in our hospital from January 2007 to November 2015 were enrolled in this retrospective research. A total of 426 males and 248 females for known or suspected SBDs were investigated with DBE, at a mean age 51.5 ± 16.6 years, range 11–88 years. All of the 15 failed DBE procedures had been excluded, in 5 of which we were unable to progress in the terminal ileum via the anal route, whereas in the other 10 patients there was poor bowel preparation or the procedure was not tolerated. The indications included the following: obscure gastrointestinal bleeding (OGIB) in 247 cases, abdominal pain in 200 cases, chronic diarrhea in 66 cases, intestinal obstruction in 58 cases, abdominal distention in 22 cases. The other 81 cases involved weight loss, anemia, nausea and vomiting, fever, and so on. Among them, patients were divided into 3 groups by age, 308 patients in the young group (11–44 years), 272 patients in the middle-aged group (45–65 years), 94 patients in the elderly group (66–88 years). Table 1 listed the patient characteristics. Data retrospectively collected included demographics, clinical, endoscopic findings, complications, diagnostic yield, and management.

**Table 1:**
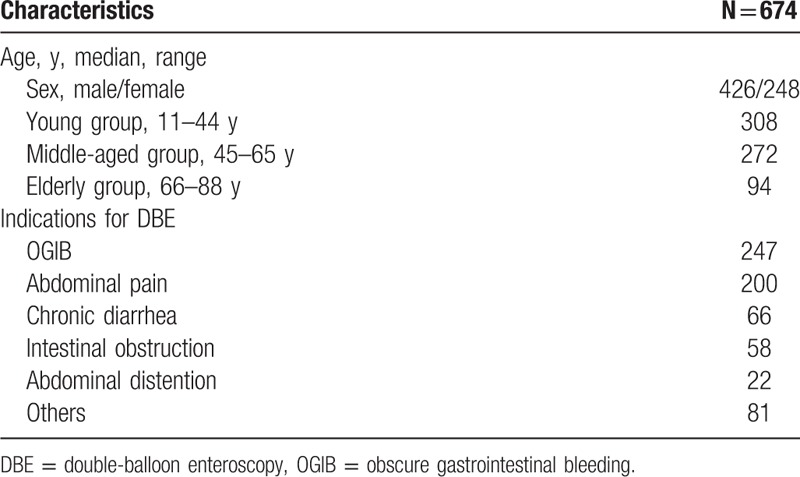
Patient characteristics.

### 2.2 DBE system and procedure

Examinations of the small bowel were performed with the DBE system (EN-450P5 or 450T5; Fujinon, Inc, Saitama, Japan). The EN-450P5 is a diagnostic type of endoscope, and the EN-450T5 is a therapeutic type of endoscope. Briefly, the operating system consisted of a mainframe, a 200 cm long enteroscope, a 145 cm long overtube and an air pump. Two latex balloons are attached to the tip of the endoscope and the overtube which can be inflated and deflated with air by a pressure-controlled pump system. Olive oil and water were added as lubricants to the space between enteroscope and the overtube to reduce the friction during the operation. We both used the same standard technique of guiding the scope in the small bowel with sequential inflation and deflation of the balloons for the “push and pull back” maneuver described by Yamamoto et al.^[5]^ DBE was performed via the oral, anal or both approaches determined by the estimated location of the suspected lesions. When the location was uncertain, the oral approach was preferred. The whole procedure was performed through the cooperation of 2 doctors and 1 nurse. The examination continued until the target lesion was reached, or until no further progress was deemed possible.

### 2.3 Preoperative preparation

A low residue and liquid diet were required and colored foods were avoided the day prior to the test. Preparation for the oral route included only fasting overnight, whereas for the rectal route bowel cleansing was required as in colonoscopy. We used 3 boxes of polyethylene glycol electrolyte (69.56 mg × 3) diluted in 3000 mL of water 5 to 6 hours before the examination. The DBE was performed with the patient under conscious or deep sedation administered by the anesthesiologist. Conscious sedation required the intravenous injection of midazolam and meperidine. General anesthesia was indicated for selected patients who were administered a combination of intravenous propofol and fentanyl. Tracheal cannula was needed in patients via the oral approach with deep sedation. During DBE, oxygen was inhaled with electrocardiographic monitoring.

### 2.4 Statistical analysis

All statistical analyses were performed with SPSS version 16.0 for Windows. Continuous data were presented as means, mean ± SD or range, and categorical variables were expressed as frequency or percentages. Qualitative variables were compared using chi-square testing. Fisher's exact probability was used when the theoretical frequency was <5. A *P* of < 0.05 was considered statistically significant.

### 2.5 Ethics statement

We obtained human subjects approval from Ethics Committee of The First Affiliated Hospital, College of Medicine, Zhejiang University. Patient records/information was anonymized and de-identified prior to analysis. Our institutional review board (Reference number 2016–229) approved this study with a waiver of informed consent.

## 3 Results

Over a period of 8 years, 744 consecutive DBE procedures were performed in our hospital. Fifteen failed cases (1 antegrade and 14 retrograde) were excluded in our study; the remaining 729 DBE procedures (397 antegrade and 332 retrograde procedures) performed in 674 patients were included in this series. A total of 55 cases underwent both via oral and anal approaches. Two patients accepted the oral approach completed the whole small bowel examination. Generally, we judged the approximate location through the inserted depth of the endoscope, the size of the enteric cavity, and the shape of the mucosal fold and villi. Table 2 shows the findings of patients undergoing DBE at our center.

**Table 2:**
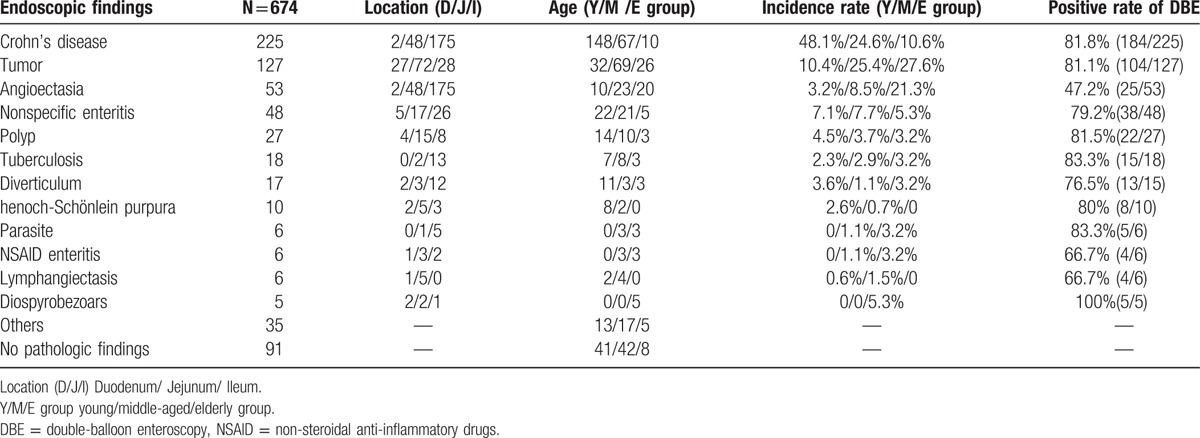
DBE findings.

### 3.1 Safety of DBE

In this series, complaints of discomfort such as sore throat, nausea, abdominal distension and abdominal pain, occurred commonly in most cases during the examination like in the gastroscopy and colonoscopy examination. However, these symptoms were transient and tolerable. Most procedures were successfully performed without severe complications, except for 3 patients with perforation, 2 patients with postprocedural hemorrhage, and 1 patient with aspiration pneumonia. The overall complication rate was 0.96% (6/729). Only 2 complications occurred in the therapeutic procedures. No acute pancreatitis or other major complications occurred.

### 3.2 DBE findings

**Figure 1:**
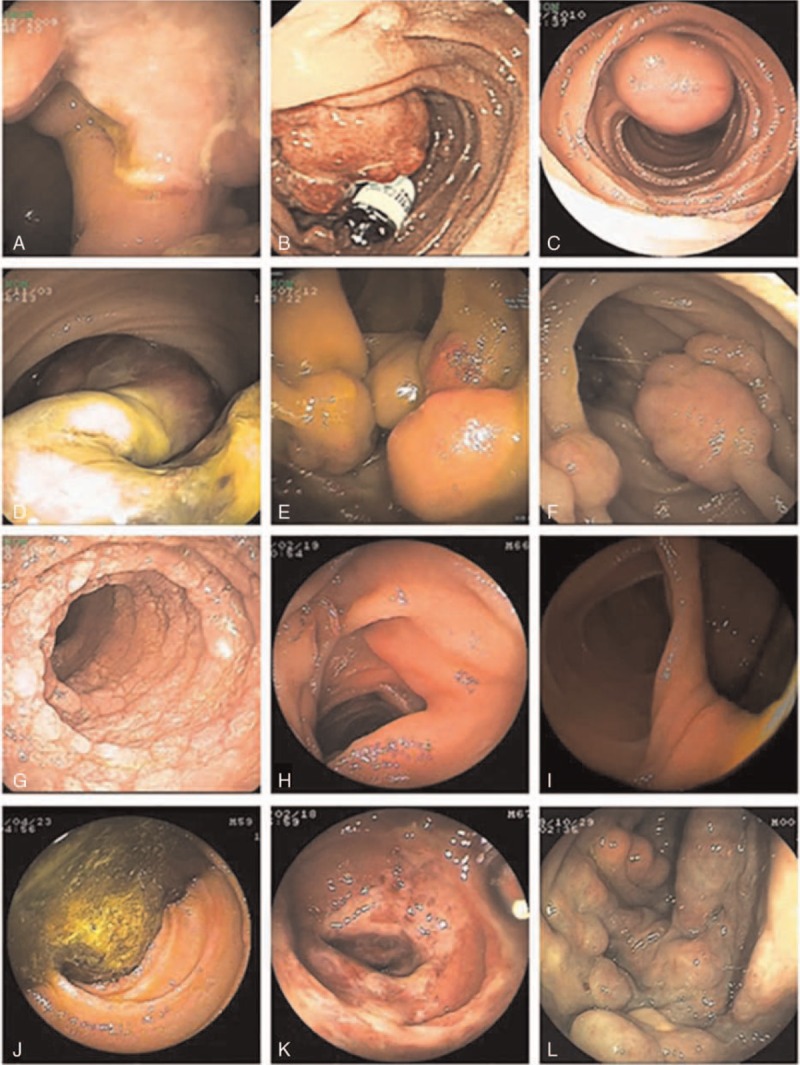
Typical gastrointestinal imaging: (A) Crohn's disease and Sinus tracts, (B) adenocarcinoma with the capsule retention, (C) GIST, (D) hamartoma, (E) vasculolymphatic tumor, (F) Peutz–Jeghers syndrome, (G) lymphangiectasis, (H) parasite, (I) diverticulum, (J) diospyrobezoars, (K) henoch-Schönlein purpura, (L) Blue Rubber Bleb Nevus Syndrome.

More than 20 types of SBDs were found in the total of 729 DBE procedures with a detection rate of abnormal findings at 70.9%(517/729). Some with negative DBE results could be finally diagnosed through other procedures, such as CE, operation, digital subtraction angiography (DSA), CT enterography (CTE), and so on. Ultimately, 91 patients had no abnormal findings. The majority of findings were Crohn's disease (33.4%, 225/674), followed by tumor (18.8%, 127/674) and angioectasia (7.9%,53/674). Other frequent DBE findings were as follows: nonspecific inflammation (48 cases), polyp (27 cases), tuberculosis (18 cases), diverticulum (17 cases), Henoch–Schönlein purpura (10 cases), parasite (6 cases), non-steroidal anti-inflammatory drugs (NSAID) enteritis (6 cases), lymphangiectasis (6 cases), diospyrobezoars (5 cases). In addition, some rare diseases such as ischemic enteritis, Dieulafoy lesions, abdominal cocoon, blue rubber bleb nevus syndrome, sjogren syndrome and systemic lupus erythematosus involving the small bowel, celiac disease, and so on, were also detected in our study. Some typical endoscopic images are shown in Fig. 1.

### 3.3 Endoscopic treatment during DBE

Endoscopic treatment was performed in 60 patients in which hemostasis (17, 28.3%) and polypectomy (15, 25%) were the predominant form of intervention used. In our group, hemostasis was performed using argon plasma coagulation (APC) in 6 cases and hemoclip in 11 cases. Endoscopic mucosal resection (EMR) and endoscopic nylon cord ligation were carried out in 6 patients and 5 patients, respectively. Endoscopic foreign bodies removal was performed in 9 patients which contained 4 retained capsule endoscopy cases and 5 diospyrobezoars. In total, 8 patients who had a diagnosis of tumor by DBE had the titanium clip location before the surgery. Endoscopic biopsy was performed in 305 DBE procedures (41.8%, 305/729). The data were listed in Table 3.

**Table 3:**
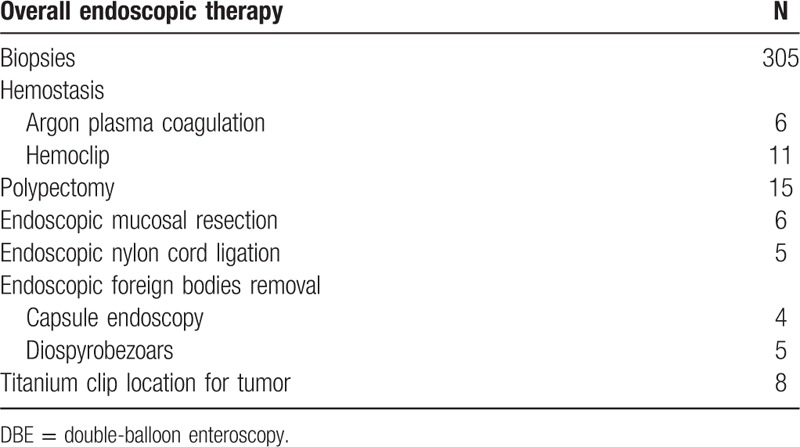
Endoscopic treatment during DBE.

### 3.4 Subgroup analysis

#### 3.4.1 The mainly SBDs detected

About 225 cases of Crohn's disease were detected in our research; the detection rate in the ileum (77.8%) was higher than duodenum and jejunum. A total of 128 tumors was identified in the patients, adenocarcinoma (38 cases), GIST (24 cases), and lymphoma (23 cases) were the most common detected. The majority of benign tumors were lipomas (7 cases). Tumors located in the jejunum had the highest detection rate (56.7%, 72/127) . Most of the tumors, such as adenocarcinoma (65.8%), lymphoma (60.9%), GIST(58.3%), and lipoma (85.7%) had a high incidence rate in the jejunum. The detection rate of angioectasia was also higher in the jejunum (54.7%). There was statistically significant difference in the positive rate of DBE between Crohn's disease (81.8%) vs angioectasia (47.2%) and tumor (81.1%) vs angioectasia (*P* < 0.05).

### 3.5 DBE in different age groups

Data were arranged in 3 different groups of age (<45, 45–65, and >65 years old). In the young cohort, Crohn's disease was the most commonly diseases followed by tumor and nonspecific enteritis. In contrast in the elderly group, the most common diagnosis was tumor, whereas angioectasia was also detected frequently. The overall diagnostic yield of DBE was 73%, 71.2%, 78.6% in young/middle-aged/elderly group. No statistically significant difference was found among them. Associated with the advancing age, the morbidity of tumor, angiodysplasia, tuberculosis, parasite, NSAID enteritis, diospyrobezoars, appeared to be increasing. Nevertheless, a decline occurred in the morbidity of Crohn's disease, polyp and Henoch-Schönlein purpura.

### 3.6 Comparison between DBE and CE

Altogether, 268 patients underwent CE examination before or after DBE; capsule retention occurred in 6 patients. The positive rate of CE was 75.4 %(202/268) which was a little higher than DBE (67.9%, 182/268). However, there was no significant statistical significance between them (*P* > 0.05). With the combinations of DBE with CE, the positive findings rate could reach 92.5 %(248/268) which was significantly higher than DBE or CE alone (*P* < 0.05). In the 225 cases of Crohn's disease, 74 patients received the CE examination with the incidence of capsule retention 6.7% (5/74), the detection rate of CE was 92.7% which was higher than DBE (81.8%, *P* < 0.05). A total of 40 cases of small bowel tumors had the CE examination with the detection rate of 84.6%, it's nearly to DBE (81.1%, *P* > 0.05). The detection rate of CE was also higher than DBE in the patients of angioectasia (69.8%vs 47.2%) (*P* < 0.05).

### 3.7 DBE in different symptoms

The most common indication for DBE was OGIB (N = 247, 36.6%); the next common indication was abdominal pain (N = 200, 29.7%). Diarrhea (N = 66, 9.8%) and intestinal obstruction (N = 58, 8.6%) were also common symptoms. Other symptoms included abdominal distention, weight loss, anemia, nausea and vomiting, fever, and so on. In our research, OGIB was most commonly caused by small bowel tumor (24.9%), followed by Crohn's disease(20.9%) and angiodysplasia(19.2%). In patients with abdominal pain, Crohn's disease (61.8%) was the mainly etiology. In all these patients, the positive rate of DBE was >70%, 71.8% in OGIB, 72.2% in abdominal pain, 84.1% in diarrhea, 76.5% in intestinal obstruction.

## 4 Discussion

A new era has been created for the diagnosis of SBDs since the development of CE and DBE in the mid-gut. Due to its great advantages, such as the direct visualization of the whole small bowel mucosa, biopsy retrieval and therapeutic interventions, DBE has been widely used in clinical practice worldwide for nearly 15 years. The advances in evolving technology and the experience of the clinicians have made this particularly difficult procedure highly effective and safe for evaluation of the small bowel. In this article of our retrospective single-centre study, we present a large cohort of consecutive patients examined by DBE during a period of 8 years.

Previous reports demonstrated a diagnostic yield for DBE ranging from 43% to 81%.^[6]^ Our retrospective chart review was conducted in 729 consecutive DBE procedures with the positive rate of 70.9% which was similar to the literature. Our overall complication rate for DBE was 0.96%, which compares favorably with previously published complication rates for diagnostic DBE(0.4–0.8%) and therapeutic DBE(3–4%).^[7]^ The adverse events included perforation (3 patients), hemorrhage (2 patients), and aspiration pneumonia (1 patient). No acute pancreatitis or other major complications occurred. In Saygili^[8]^ et al reports, patients who had previous abdominal surgery and altered anatomy had greater risk of complications. Experience of the endoscopist, shorter time of procedure, and inflating the balloons distal to Treitz ligament were the clues to reduce the rate of complications. In our group, DBE were also performed in patients >80 years and <20 years with no raised complication rate. Cangemi et al^[9]^ also reported age alone should not be a contraindication to perform DBE when clinically indicated. All of this suggested that DBE was a safe, well-tolerated procedure for the diagnosis of SBDs in different age groups.

In our study, Crohn's disease, tumor and angioectasia ranked as the top 3 positive findings among patients with suspected SBDs. The comparison of findings as compared to the literature demonstrated significant regional variation—inflammatory lesions and tumor were the most common positive finding in Asian populations, whereas in Western countries, vascular lesions accounted for the majority.^[10]^ The diseases distribution was also different in different age groups. Crohn's disease was the most common disease followed by tumor and nonspecific enteritis in young patients; however, in the old age cohort, the most common finding was tumor, whereas angioectasia was also detected frequently. There was no statistically significant difference in the diagnostic yield of DBE of different groups. The morbidity of tumor, angiodysplasia, tuberculosis, parasite, NSAID enteritis, diospyrobezoars, appeared to be increasing along with the advancing of age. In contrast, a decline of the morbidity occurred for Crohn's disease, polyp and henoch-Schönlein purpura. As shown in our series, hemostasis and polypectomy occupied the majority of endoscopic therapy. APC and hemoclip were the main forms of hemostasis. In addition, endoscopic nylon cord ligation, endoscopic foreign bodies removal, EMR, were also performed in our series.

Crohn's disease was the most frequently SBD in the whole cohort, 77.8% of which was located in the ileum. The positive rate of DBE in these Crohn's disease patients was about 81.8%. Adenocarcinoma, GIST, and lymphoma were the most common tumor detected and the majority of these tumors were located in the jejunum, which is consistent with the literature.^[11]^ Landry et al^[12]^ reported that most of the small bowel tumors occurred primarily in the proximal small bowel (duodenum and jejunum) except for lymphomas, sarcomas, and carcinoids. The detection rate of angioectasia was also higher in the jejunum (54.7%) with DBE positive rate of 47.2% which was lower than Crohn's disease and tumor. The location of the lesion can guide the insertion route of DBE; therefore in our hospital, when the ancillary tests could not identify the location, the transoral approach was chosen in patients suspected of tumor and transanal approach in patients suspected of Crohn's disease. Generally, the oral route may be preferred as a first choice in most of the patients. Insertion depth using the oral route was higher, 270 cm beyond Treitz ligament and 150 cm proximal to ileocecal valve were considered as the average insertion depths for oral and anal route respectively according to the literature.^[13]^ Meanwhile, the retrograde procedure was recognized as technically more challenging. Maximal depth of insertion was significantly influenced by history of abdominal-pelvic surgery, insertion route, gender and type of enteroscope used.^[14]^ In our series, 2 patients selected for the oral approach had the tube inserted up to the ascending colon which was rare in the reports.

The introduction of DBE and CE has revolutionized the way that the small bowel is investigated and treated, both of whom have their own advantages and limitations. In total 268 patients underwent the examination of CE with a capsule retention rate of 2.2%. In everyday practice, the overall incidence of capsule retention was estimated to be rather low (∼1–2%) with two-thirds of the retention cases being secondary to CD-related strictures.^[15,16]^ In our research, The detection rate of CE in Crohn's disease and angioectasia was higher than DBE; however, capsule retention needed your consideration which was about 6.7% in our patients of Crohn's disease. Overall, like the previous studies reported,^[17]^ CE and DBE have demonstrated comparable diagnostic yields. Nevertheless, the combination of them may significantly improve the detection rate and diagnostic accuracy of SBDs. In most clinical scenario, CE was the initial approach and after addressing the site of pathology, DBE was used for sampling or therapeutic intervention. The route for DBE could be also determined according to the outcome of CE.^[18]^ So, the capsule-directed DBE procedure has been widely accepted in developed countries.^[19]^ Nevertheless, the high price confined the indication of this sequential approach in our country. DBE could take the first place instead of CE in patients with severe and persistent bleeding or the probability of intervention was strong, patients with obstruction.

In large-sample studies, OGIB was the leading indication for DBE, and the diagnostic yield for OGIB was 43% to 75%.^[20]^ Not surprisingly, the leading indication for our DBE series was also OGIB (36.6% of patients). As shown in our series, lesions may be missed on conventional endoscopy which included 3 cases of duodenal ulcer and 1 case of Dieulafoy lesion who had OGIB. Missed nonsmall-bowel lesions (NSBLs) have been a problem previously reported in many published series which could account for up to 24.6%.^[21]^ Suboptimal cleansing, inaccurate examination time, small lesions in atypical locations, procedures performed too quickly were possible explanations for the missed lesions.^[22]^ A careful repetition of the examination with gastroscopy and colonoscopy might be required. Abdominal pain, diarrhea, and intestinal obstruction were the other common indications for DBE. The diagnostic yield was equally high in these patients, 72.2% in abdominal pain, 84.1% in diarrhea, and 76.5% in intestinal obstruction respectively. All of the data implied the high diagnostic value of DBE for different symptoms.

Our study also had its limitations. Firstly, although there were large sample sizes of patients in our research, this study was only single center study, the selection of patients may have been biased in many aspects. Secondly, patients with endoscopic treatment relatively few compared to diagnostic DBE.

In summary, our study showed that DBE is a useful diagnostic and therapeutic tool with high clinical practice value for the investigation of SBDs. The procedure is also safe and well tolerated in young and elderly patients in the same fashion as in adult patients. Nowadays, DBE is now being offered routinely worldwide in tertiary centers. Procedural complexity, moderate complication rate, and long procedure time are obstacles to incorporating DBE into daily practice.^[23]^ With growing experience of the endoscopist, we believe that DBE must be kept in mind as the first diagnostic and therapeutic procedure for suspected SBDs.
